# *Mycobacterium tuberculosis* sensu stricto in African Apes, What Is Its True Health Impact?

**DOI:** 10.3390/pathogens11050484

**Published:** 2022-04-19

**Authors:** Carlos R. Sanchez, Ezequiel Hidalgo-Hermoso

**Affiliations:** 1 Veterinary Medical Center, Oregon Zoo, Portland, 4001 SW Canyon Rd., Portland, OR 97221, USA; 2 Conservation and Research Department, Parque Zoologico Buin Zoo, Panamericana Sur Km 32, Buin 01730, Chile; ezequielhidalgovet@yahoo.com

**Keywords:** chimpanzee, gorilla, tuberculosis, apes, zoos, African

## Abstract

Since the Symposium on Mycobacterial Infections of Zoo Animals held at the National Zoological Park, Smithsonian Institution in 1976, our understanding of tuberculosis (TB) in non-domestic animals has greatly expanded. Throughout the past decades, this knowledge has resulted in improved zoo-habitats and facilities design, stricter biosecurity measures, and advanced diagnostic methods, including molecular techniques, that have significantly decreased the number of clinical disease caused by *Mycobacterium tuberculosis* in apes under human care settings. In the other hand, exponential growth of human populations has led to human encroachment in wildlife habitat which has resulted in increased inter-species contact and recurrent conflict between humans and wild animals. Although it is widely accepted that non-human primates are susceptible to *M. tb* infection, opinions differ with regard to the susceptibility to develop disease amongst different taxa. Specifically, some authors suggest that African apes are less susceptible to clinical tuberculosis than other species of primates. The aim of this review article is to evaluate the current scientific literature to determine the actual health impact of disease caused by *Mycobacterium tuberculosis* and more specifically *Mycobacterium tuberculosis* sensu stricto in African apes. The literature review included literature databases: Web of Science, Pubmed, Scopus, Wiley, Springer and Science direct, without temporal limit and proceedings of annual conferences in the field of wildlife health. Our general inclusion criteria included information about serological, molecular, pathological (macroscopic and/or microscopic), and clinical evidence of TB in African apes; while our, our more stringent inclusion selection criteria required that in addition to a gross pathology, a molecular test confirmed *Mycobacterium tuberculosis* sensu stricto as the cause of disease or death. We identified eleven reports of tuberculosis in African apes; of those, only four reports met the more stringent selection criteria that confirmed *M. tb* sensu stricto in six individuals. All reports that confirmed *M. tb* sensu stricto originated from zoological collections. Our review suggests that there is little evidence of disease or mortality caused by *M. tb* in the different species of African apes both under human care and free ranging populations. Additional studies are needed in free-ranging, semi-captive populations (sanctuaries) and animals under human care (zoos and rescue centers) to definitely conclude that this mycobacteria has a limited health effect in African ape species.

## 1. Introduction

Since the Symposium on Mycobacterial Infections of Zoo Animals held at the National Zoological Park, Smithsonian Institution in 1976 [1] our understanding of tuberculosis (TB) in non-domestic animals has greatly expanded. Tuberculosis (TB) is the most impactful bacterial disease to affect humanity, with a quarter of all humans infected [2,3]. It is responsible for about 10.4 million new cases per year and 1.5–2 million deaths around the world [4]. Although TB remains a global challenge, cases are highly concentrated in very specific parts of the world, with nearly 90% of all human TB cases located in South Asia, East Asia (China), South East Asia (Philippines, Indonesia) and, the most populous countries in Africa [2].

In mammals, tuberculosis is caused by bacteria members of the *Mycobacterium tuberculosis* complex (MTBC) in the *Mycobacteriaceae* family [5]. Mycobacteria are aerobic, slightly curved or straight, occasionally beaded, rod-shaped bacilli that stain poorly with Gram’s stain, but stain positively with acid-fast stains (e.g., ZiehleNeelsen, FiteeFaraco) [6]. Genome studies show that MTBC comprises: (1) five human-adapted lineages, Lineage 1–4 and L7 represent *M. tuberculosis* sensu stricto, (2) two other human Lineages (L5 and L6) represent *M. africanum* and (3) a minimum of 9 animal-adapted lineages [7,8,9,10].

In non-human primates (NHPs) the majority of TB cases reported are caused by *Mycobacterium tuberculosis* sensu stricto (from here on *M. tb*) [11,12,13]. More recently, a novel *Mycobacterium tuberculosis Complex* strain was isolated from a wild Chimpanzee killed by a leopard in Cote d’Ivoire; the authors proposed to name it “Chimpanzee Bacillus” rather than to designate a new species or subspecies name [14]. *M tb* can also affect domestic species [15,16,17,18] and other wildlife species [19,20,21]. 

Humans are the main reservoirs host of *M. tb* [22,23] and infection in NHPs mostly occurs by close contact with TB-infected humans through the inhalation of aerosolized bacteria or other routes, such as ingestion and wounds [13,19]. Conversely, there is no evidence of direct primate-to-human transmission [24]. Clinical presentation of tuberculosis in apes can be divided into symptomatic TB and latent infections; only symptomatic animals can transmit tuberculosis. Animals with latent TB may appear healthy for years, but eventual reactivation of latent TB can result in secondary transmission and outbreaks of disease in established colonies [12]. Both in humans and non-human primates this is a chronic disease and clinical signs are non-specific including weight loss, anorexia, and poor body condition; in more advanced cases respiratory signs such as dyspnea and coughing can be present [23,25]. *M. tb* causes progressive pulmonary disease in many non-human primate species [26]. As it occurs in humans, the respiratory system is the primary site of infection, with involvement of lungs and local lymph-nodes; in advanced cases systemic spread to liver, kidneys, spinal column, spleen and other organs can occur [11,23]. When the respiratory tract is the primary site of infection, post-mortem lesions consist of multifocal to coalescing granulomatous pneumonia. Oral infections are characterized by granulomatous lesions in the abdominal viscera and the mesenteric lymph-nodes [11,23]. Since lesions are similar among the different members of the MTBC, PCR testing is needed to definitely confirm infection by *M. tb* [23].

All non-human primates are susceptible to *M. tb* infection, but expert’s opinions diverge over the susceptibility to develop disease among primate species. Several authors [4,11,24,25,27,28] affirm that African apes (*Gorilla gorilla, Gorilla beringei, Pan paniscus, Pan troglodytes*) are less likely to develop disease and to die of TB than other primates. Conversely, African green monkeys (*Chlorocebus aethiops*) and macaque species appear to be the most susceptible to tuberculosis [11,12,24,29]. The objective of this review is to evaluate the scientific literature to provide current evidence-based information about the health impact of *Mycobacterium tuberculosis* sensu stricto in African apes. Systematic review of the scientific literature will be used to summarize relevant evidence with regard to this topic.

## 2. Material and Methods

The literature review was executed during September and November 2021.

### 2.1. Eligibility Criteria

Articles and reports included for revision met the following criteria:

(1) Only literature in English was considered
(2) Full online availability for a thorough review
(3) Included captive and free-ranging African ape species (*Gorilla gorilla, Gorilla beringei, Pan paniscus, Pan troglodytes*) without specific geographical location.
(4) Included information about serological, molecular, pathological, and clinical evidence of TB in African apes.
(5) Included information about suspected TB cases (clinical presentation and/or pathologic [gross] lesions) and confirmation of *Mycobacterium tuberculosis* sensu stricto by molecular testing. Diagnosis of TB in apes, pre or post mortem, is not straightforward [30,31]. Due to this conundrum, our more stringent inclusion selection criteria required that in addition to a pathologic gross diagnosis, a microbiological or molecular test confirmed *Mycobacterium tuberculosis* as the cause of disease or death [23,32].
(6) The reports have been published in peer-reviewed journals and/or proceedings for the annual conferences of the American Association of Zoological Veterinarians (AAZV), the Wildlife Diseases Association (WDA), or the European Association of Zoo and Wildlife Veterinarians (EAZWV) from 1970–2020, 1996–2021 and 2013–2019, respectively. Proceedings were included because of their online availability at the time of the literature search. Furthermore, the proceeding inclusion allowed us to include TB reports that were never published in a peer-reviewed journal.

### 2.2. Information Sources

The following scientific databases were used to systematically search and gather the articles from where the information will be extracted:

- Web of Science (https://clarivate.com/products/web-of-science accessed on 5 September 2021)
- PubMed (https://pubmed.ncbi.nlm.nih.gov/ accessed on 5 September 2021)
- Scopus (https://www.scopus.com/ accessed on 5 September 2021)
- Wiley (https://onlinelibrary.wiley.com accessed on 1 October 2021)
- Springer (https://www.springer.com accessed on 1 October 2021)
- Science direct (https://www.sciencedirect.com accessed on 1 October 2021)
- Articles were evaluated regardless of their publication date.

### 2.3. Search Strategy

The following keywords were used to search for publications in the three information sources indicated above:

- Tuberculosis AND Chimpanzee
- Tuberculosis AND Apes
- Tuberculosis AND Gorilla
- Tuberculosis AND *Pan paniscus*
- Tuberculosis AND *Pan troglodytes*
- *Mycobacterium tuberculosis* AND Apes
- *Mycobacterium tuberculosis* AND Gorilla
- *Mycobacterium tuberculosis* AND *Pan paniscus*
- *Mycobacterium tuberculosis* AND *Pan troglodytes*
- *Mycobacterium tuberculosis* AND Chimpanzee.

For each individual search performed with a specific keyword, the name of the database consulted, the total number of publications found, and the number of selected publications that fulfilled the selection criteria were recorded. After the initial search was completed on all databases, the duplicated articles were consolidated as one.

For each search carried out with a specific keyword, information about the database used, total number of publications found in that particular search, and number of selected publications was recorded in an Excel sheet.

### 2.4. Data Collection

The information extracted from scientific publications was organized and managed in Excel with the following information: (1) Animal species, (2) year of publication, (3) number of animals involved in the report, (4) sex, (5) age, (6) proposed source of infection (7) geographic location, (8) clinical presentation, (9) pathologic findings, (10) diagnostic techniques including molecular characterization if available and (11) author.

## 3. Results

We identified eleven reports of tuberculosis in African apes (Table 1), of those only four reports, describing a total of 6 cases, met the second selection criteria confirming *M. tb* [33,34,35,36]. Of the other seven reports, all but one [14] describe African apes under human care; the majority of them do not provide information about the source of infection, any ancillary tests performed or information about the mycobacteria strain involved; as a result a final diagnosis of tuberculosis caused by *M. tb* was not possible.

We were unable to find any report on bonobos. The reports with confirmed *M. tb* comprise two gorillas and 4 four chimpanzees and spanned from 1998 and 2011 and described cases only in animals under human care, specifically in zoological collections. Three of the reports originated in the African continent while one chimpanzee report originated from an Australian zoo. Of the 4 individuals where age was available, 3 out of 4 were adult animals, 1 was a juvenile chimpanzee. The age of the affected chimpanzees ranged from 5 to 24 years, 1 gorilla was 47 y.o. The age of the remaining gorilla was not specified. In 2 of the reports the visiting public was the suspected source of infection; in the case of the chimpanzee in Australia, an Asian elephant was deemed the source of infection. The remaining report did not speculate on the infection source. All cases used culture and molecular techniques to reach a final diagnosis.

## 4. Discussion

Apes, similar to humans, could be affected by different mycobacteria, nevertheless, we focused our review on *M. tuberculosis* sensu stricto based on its relevance on pubic heath worldwide and also because infection with other mycobacteria (*M*. *africanum*, *microti* and *canettii*) is considered rare in apes [28]. A detailed review of other MTBC in African apes is beyond the scope of this article.

We acknowledge that apes, similar to other NHPs, are susceptible to infection and disease by *M. tuberculosis*; however, based upon the few reports we found in the literature confirming *M. tb* as the cause of death, we can only wonder whether it is true that apes could be less susceptible to tuberculosis when compared with other primates [11,19,39]; and whether we should consider TB, caused by *M. tb,* a primary a disease of humans.

In spite of an extensive literature search spanning several decades, we found only a handful of reports of mortality caused by tuberculosis; when applying more stringent selection criteria only four reports fulfilled this criteria. A reasonable explanation for the latter, is that molecular techniques are relatively new and as such, the number of reports in African apes could have been grossly underestimated. On the other hand, it is also plausible that because the absence of PCR and genome sequence analysis, some of the cases attributed to MTBC in the past were caused by other mycobacteria (or even other infectious agents such as *Nocardia*) and the number of older reports was overestimated. We expect that next-generation sequencing technologies when applied to mycobacterial research will increase our knowledge about risk, susceptibility and mortality of *M. tb* in apes.

We recognize that by including reports only in the English language, cases reported in other languages could have been omitted. However, it remains a real possibility that there are few reports because the disease is rare both, in free ranging and animals under human care.

### 4.1. Free Ranging Populations

Our review discovered no reports of disease caused by *M. tb* in free ranging populations of African apes and similarly to what occurs in other non-human primates it appears to be mainly a problem of captive animals exposed to infected humans [27,28,36,40,41,42]. Tuberculosis is considered a major global human health concern. In equatorial Africa, where most of the remaining free-living great ape populations live, human populations and their domestic animals are considered exposed or infected by this organism [43]. As a result, it would be logical to assume that tuberculosis is also a significant health concern for wild ape populations in range countries.

An excellent review on the risk of MTBC in free ranging great apes was published in 2014 [43] and the reader is referred to it for a detailed explanation of risk, susceptibility and mortality and the relation to each other in the context of *M. tb* in apes.

The wild populations of mountain gorillas in Virunga National Park (Democratic Republic of Congo [DRC]), Volcanoes National Park (Rwanda) and Bwindi Impenetrable National Park (Uganda), and chimpanzees in Gombe National Park in Tanzania and Tai National Park in Ivory Coast, have maintained systematic health monitoring programs for well over 20 years. These programs consist of veterinary health evaluations as well as mortality investigation including screening for potential infectious agents [44,45,46,47]. These programs have shown evidence of clinical disease caused by *Mycobacterium leprae* [48] and granulomatous lesions in organs caused by a MTBC organism known as *M. africanum* West Africa 2 or *Bacillus Chimpanzee* in a chimpanzee [14], however, no reports of disease caused by *M. tb* were found in apes screened using molecular techniques. Interestingly, in the *Bacillus Chimpanzee* report, the authors investigated the possible presence of MTBC in other chimpanzees, including animals form the area and in the same community as the index case. They found no positive results in 115 archived tissue samples from 28 animals within the previous 10 years. The researchers tested tissues by rtPCR in addition to gross lesions and histological evaluation [14].

Likewise, two different studies, one evaluating 130 alive and 11 dead chimpanzees in the Kasekela community in Gombe National Park Tanzania, did not found evidence of pathological lesions associated to *M. tb* as the cause of death [49,50]. Furthermore, a recently published manuscript describing gastro-intestinal lesions of deceased mountain gorillas [51] did not reveal any evidence of tuberculosis in 60 individuals from Uganda, Rwanda, or the DRC, that died between 1985 and 2007. Whether the authors found tuberculosis in the respiratory tract is unknown, however if they did, their report will add invaluable information to our current knowledge of the prevalence of *M. tb* in this highly endangered ape. Another retrospective study of 103 deceased infant gorillas (less than 3.5 years old) of one population failed to reveal any evidence of tuberculosis over 46 years [37,50]. In this case, considering tuberculosis a chronic disease, the age of the investigated animals most likely biased the negative results. An epidemiologic study evaluating fecal samples of 91 bonobos (*Pan paniscus*) from 2 populations in a semi-captivity setting that were reintroduced into the wild at the DRC, showed no evidence of *Mycobacterium* spp. [52]. Fecal screening has low sensitivity because requires the mycobacteria to be excreted in feces, which only occurs during active infection. A limitation of this non-invasive screening technique is that animals with latent infection are not detected and as a result some of the results could have been false negatives. Similarly, screening for *M. tb* in free-ranging chimpanzees [53] did not find evidence of *M. tb* in PCR testing of feces from 68 individuals living in Gombe National Park, Tanzania. The researchers clearly expressed the limitations of fecal screening in their study; nevertheless, they were able to conclude that they did not identify any individuals with active MTBC infection [53].

A plausible explanation for the lack of reports of clinical TB in wild chimpanzees is that their life expectancy in the wild is about 15 years, which is considerably shorter than the life span reported for chimpanzees in zoos or captive breeding colonies [54,55]. This short life-span would not allow animals to develop clinical signs of this chronic disease in this species, however, we would have expected that screening of deceased animals would have shown at least some evidence of mycobacteria lesions even in asymptomatic animals.

It is important to recognize that the use of molecular techniques for the diagnosis of *M. tb* is not routinely used in some of the areas with the highest prevalence for this pathogen. As a result, the low number of reports of *M. tb* in apes could simply reflect that the number of cases is underestimated.

Another potential, and perhaps a controversial assertion of this review, is that at this time, *M. tb* may not seem to represent a significant threat to the health or conservation of wild African ape species; at least not at the same degree as other infectious pathogens such as anthrax, Ebola, simian immunodeficiency virus and respiratory viruses [56,57,58,59,60,61,62,63,64]. Conversely, some authors consider that small outbreaks could have a strong effect in the viability of isolated populations in great apes [14]; what is certain is that more work is needed to determine the health effect and susceptibility of *M. tb* infection in great ape species.

Finally, one additional reason to explain the lack of reports in wild apes is that there does not appear to exist an intraspecific infection in apes. It is reasonable to think that humans are the source of infection for wild populations through direct contact between researchers, visitors, local people, etc., and apes [55,65]. Due to this, it is important to continue implementing and enforcing sanitary protocols for visitors, researchers and any other personnel with potential direct contact with these species. These protocols have proven to be effective in reducing the risk of pathogen exposure in the mountain gorilla and chimpanzee habitats [47,66] which is likely reflected in the absence of tuberculosis reports in these species.

### 4.2. Animals under Human Care

As shown in Table 2, the reports of disease by *M. tb* originated in zoos which are environments where close contact between infected humans and animals might allow for aerosol transmission of the bacterium [67,68]. It is generally considered that the number of cases of *M. tb* in animals under human care has decreased in the past 40 years [12,23]. The low number of reports we found in African apes under human care in our review confirm this assertion. A search of the archives at a large private zoo pathology service based in the U.S.A, from 1994–2021 identified only 1 potential case in a chimpanzee. This case was diagnosed in 1999 as tuberculosis suspicious based on lesion morphology and distribution, and the presence of very low numbers of acid-fast bacilli in the lesion, no cultures were performed and molecular techniques were not available at the time; as such confirmation of tuberculosis by *M. tb* was not feasible (Garner M. Northwest Zoo Path. Pers Comm).

Likewise, in one of the most comprehensive reviews available, (ranging from 1980 to 2014) in a large primate center also in the U.S.A, the pathologic lesions from 245 chimpanzees were investigated; not a single case of TB infection was reported [71].

These findings favor the hypothesis that new exhibit design, the use of personal protective equipment (PPE) and aggressive preventive measures have driven down the prevalence of tuberculosis in zoos in the last decades.

While new exhibits limit direct contact between visitors and animals, something that occurred in the past, the use of PPE by employees including disposable gloves, rubber boots, disposable face mask and in some cases use of face shields or goggles, have limited employee exposure to animal diseases and the other way around.

An effective preventive medicine program is of paramount importance in zoological collections and in the case of ape species, include preventive measures such as: pre-shipment examinations, strict quarantine of newly imported animals and extensive testing of all new primate arrivals using TST (Tuberculin Skin Test) and screening chest radiographs; in some institutions, broncho-alveolar and gastric lavages are routinely used as screening tool for TB; the collected samples are submitted for culture and/or PCR testing. Furthermore, most zoos actively implement employee health programs where caretakers in direct contact with primates are tested for *M. tb* either by TST or an interferon (IFN)-gamma release assay (IGRA) that assesses the cell-mediated immune response to *Mycobacterium tuberculosis complex* antigens.

Recently, a Quantitative Rapid serologic test for detection and monitoring of active pulmonary TB in nonhuman primates has shown great promise in the tested macaques [72]; we should expect that in the near future, additional diagnostic tools will be available to rapidly and accurately diagnose and confirm TB in non-human primates.

A caveat to our review of available reports of TB in animals under human care is that in certain geographic areas there are no mandatory regulatory surveillance systems in place for zoological collections; as a result tuberculosis could be either underreported or not reported at all due to concerns of negative public perception [33,73].

## 5. Conclusions

In summary, our review revealed that there is little published evidence of disease caused by *M. tb* in the different species of African apes both under human care and free ranging populations. Additional studies evaluating captive and free-ranging animals in developing countries, with the aid of molecular diagnostic techniques, are needed to definitely conclude that African apes have low TB disease susceptibility to *M. tb* and that this human pathogen is not a substantial threat to the health and conservation of this species.

## Figures and Tables

**Table 1:** Reports of Tuberculosis in African apes.

Species	Year	Number	Sex	Age	Strain	Location	Ancillary Test	Ref.
*Pan troglodytes*	1932	3	2M/1F	NA	NA	Zoo of Rome	NA	[33]
*Gorilla gorilla*	1949	1	NA	NA	NA	Circus	NA	[34]
*Gorilla gorilla*	1966	1	F	Juvenile	Bovine	London Zoo	Culture	[35]
*Pan troglodytes*	1998–2000	2	NA	18 y.o 5 y.o	NA	National Zoological Gardens of South Africa	Acid fast stain (sputum/feces)	[36]
*Gorilla gorilla*	2001	3	NA	Juvenile	NA	London Zoo	NA	[37]
*Pan troglodytes*	2001	1	NA	NA	NA	Japanese Zoo	Tuberculin	[22]
*Gorilla gorilla*	2002	1	F	Adult	NA	Melbourne Zoo	IFN-γ (pos) PCR (neg) Culture (neg)	[38]
*Pan troglodytes*	2009	1	F	Adult	Bacillus Chimpanzee	Côte d’Ivoire	rtPCR	[14]

NA: Not available.

**Table 2:** Confirmed reports of *Mycobacterium tuberculosis* in African apes.

Species	Year	Number	Sex	Age	Source of Infection	Location	Ancillary Test	Ref.
*Pan troglodytes*	1991–2001	2	NA	23 y.o 5 y.o	Visiting public	National Zoological Gardens of South Africa	Culture/IS6110 RFLP	[38]
*Pan troglodytes/Gorilla gorilla*	2003–2009	1/1	F/M	18 y.o/ Adult (age not specified)	NA	National Zoological Gardens of South Africa	Culture/VNTR, RFLP	[67]
*Pan troglodytes*	2011	1	M	NA	Asian Elephant	Australian Zoo	PCR and Culture	[69]
*Gorilla gorilla*	2009	1	F	47 y.o	Visiting public	Nigerian Zoo	DNA hybridization nitrocellulose strips	[70]

NA: Not available. RFLP: Restriction fragment length polymorphism analysis. VNTR: Variable number of tandem repeat.

## Data Availability

Not applicable.

## References

[1] Griner L.A., Montali R.J. (1983). Occurrence of tuberculosis in the zoo collection of the zoological society of San Diego, 1964–175. Mycobacterial Infections of Zoo Animals.

[2] Kock R., Michel A.L., Yeboah-Manu D., Azhar E.I., Torrelles J.B., Cadmus S.I., Brunton L., Chakaya J.M., Marais B., Mboera L. (2021). Zoonotic tuberculosis—The changing landscape. Int. J. Infect. Dis..

[3] Rodriguez-Campos S., Smith N.H., Boniotti M.B., Aranaz A. (2014). Overview and phylogeny of *Mycobacterium tuberculosis* complex organisms: Implications for diagnostics and legislation of bovine tuberculosis. Res. Vet. Sci..

[4] Obaldía N., Nuñez M., Montilla S., Otero W., Marin J.C. (2018). Tuberculosis (TB) outbreak in a closed Aotus monkey breeding colony: Epidemiology, diagnosis and TB screening using antibody and interferon-gamma release testing. Comp. Immunol. Microbiol. Infect. Dis..

[5] The Center for Food Security and Public Health (CFSPH) (Last updated: October 2019). Zoonotic Tuberculosis in Mammals, including Bovine and Caprine Tuberculosis. Retrieved from Center for Food Security and Public Health. https://www.cfsph.iastate.edu/Factsheets/pdfs/bovine_tuberculosis.pdf.

[6] Lowenstine L.J., Osborn K.G. (2012). Respiratory system diseases of nonhuman primates. Nonhuman Primates in Biomedical Research.

[7] De Jong B.C., Adetifa I., Walther B., Hill P.C., Antonio M., Ota M., Adegbola R.A. (2010). Differences between tuberculosis cases infected with *Mycobacterium africanum*, West African type 2, relative to Euro-American *Mycobacterium tuberculosis*: An update. FEMS Immunol. Med. Microbiol..

[8] Brites D., Loiseau C., Menardo F., Borrell S., Bniotti M.B., Warren R., Dippenaar A., Parsons S.D.C., Beisel C., Behr M.A. (2018). A new phylogenetic framework for the animal-adapted mycobacterium tuberculosis complex. Front. Microbiol..

[9] Ngabonziza J.C.S., Loiseau C., Marceau M., Jouet A., Menardo F., Tzfadia O., Antoine R., Niyigena E.B., Mulders W., Fissette K. (2020). A sister lineage of the *Mycobacterium tuberculosis* complex discovered in the African Great Lakes region. Nat. Commun..

[10] Bespiatykh D., Bespyatykh J., Mokrousov I., Shitikov E. (2021). A Comprehensive map of *Mycobacterium tuberculosis* complex regions of difference. mSphere.

[11] Renquist D.M., Whitney R.A., Montali R.J. (1978). Tuberculosis in nonhuman primates-an overview. Mycobacterial Infection of Zoo Animals.

[12] Lerche N.W., Yee J.L., Capuano S.V., Flynn J.L. (2008). New approaches to tuberculosis surveillance in nonhuman primates. ILAR J..

[13] Warit S., Billamas P., Makhao N., Jaitrong S., Juthayothin T., Yindeeyoungyeon W., Dokladda K., Smittipat N., Kemthong T., Meesawat S. (2020). Detection of tuberculosis in cynomolgus macaques (*Macaca fascicularis*) using a supplementary monkey interferon gamma releasing assay (mIGRA). Sci Rep..

[14] Coscolla M., Lewin A., Metzger S., Maetz-Rennsing K., Calvignac-Spencer S., Nitsche A., Dabrowski P.W., Radonic A., Niemann S., Parkhill J. (2013). Novel *Mycobacterium tuberculosis* complex isolate from a wild chimpanzee. Emerg. Infect. Dis..

[15] Fetene T., Kebede N., Alem G. (2011). Tuberculosis infection in animal and human populations in three districts of Western Gojam, Ethiopia. Zoonoses Public Health.

[16] Hiko A., Agga G.E. (2011). First-time detection of mycobacterium species from goats in Ethiopia. Trop. Anim. Health Prod..

[17] Parsons S.D., Gous T.A., Warren R.M., van Helden P.D. (2008). Pulmonary *Mycobacterium tuberculosis* (Beijing strain) infection in a stray dog. J. S. Afr. Vet. Assoc..

[18] Jenkins A.O., Cadmus S.I., Venter E.H., Pourcel C., Hauk Y., Vergnaud G., Godfroid J. (2011). Molecular epidemiology of human and animal tuberculosis in Ibadan, Southwestern Nigeria. Vet. Microbiol..

[19] Montali R.J., Mikota S.K., Cheng L.I. (2001). *Mycobacterium tuberculosis* in zoo and wildlife species. Rev. Sci. Tech..

[20] Alexander K.A., Pleydell E., Williams M.C., Lane E.P., Nyange J.F., Michel A.L. (2002). *Mycobacterium tuberculosis*: An emerging disease of free-ranging wildlife. Emerg. Infect. Dis..

[21] Miller M.A., Buss P., Roos E.O., Hausler G., Dippenaar A., Mitchell E., van Schalkwyk L., Robbe-Austerman S., Waters W.R., Sikar-Gang A. (2019). Fatal tuberculosis in a free-ranging African elephant and one health implications of human pathogens in wildlife. Front. Vet. Sci..

[22] Une Y., Mori T. (2007). Tuberculosis as a zoonosis from a veterinary perspective. Comp. Immunol. Microbiol. Infect. Dis..

[23] Lowenstine L.J., McManamon R., Terio K. (2018). Apes. Pathology of Wildlife and Zoo Animals.

[24] Ghodbane R., Drancourt M. (2013). Non-human sources of *Mycobacterium tuberculosis*. Tuberculosis.

[25] Murphy H.W. (2015). Great Apes. Fowler’s Zoo and Wild Animal Medicine.

[26] Vervenne R.A., Jones S.L., van Soolingen D., van der Laan T., Andersen P., Heidt P.J., Thomas A.W., Langermans J.A. (2004). TB diagnosis in non-human primates: Comparison of two interferon-gamma assays and the skin test for identification of *Mycobacterium tuberculosis* infection. Vet. Immunol. Immunopathol..

[27] Isaza R., Fowler M.E., Miller R.E. (2003). Tuberculosis in all taxa. Zoo and Wild Animal Medicine.

[28] Mätz-Rensing K., Hartmann T., Wendel G.M., Frick J.S., Homolka S., Richter E., Munk M.H., Kaup F.J. (2015). Outbreak of tuberculosis in a colony of rhesus monkeys (*Macaca mulatta*) after possible indirect contact with a human TB patient. J. Comp. Pathol..

[29] Motzel S.L., Schachner R.D., Kornegay R.W., Fletcher M.A., Kanaya B., Gomez J.A., Ngai D.T.-W., Pouch W.J., Washington M.V., Handt L.A. (2003). Diagnosis of tuberculosis in nonhuman primates. International Perspectives: The Future of Nonhuman Primate Resources.

[30] Rosenbaum M., Mendoza P., Ghersi B.M., Wilbur A.K., Perez-Brumer A., Cavero Yong N., Kasper M.R., Montano S., Zunt J.R., Jones-Engel L. (2015). Detection of *Mycobacterium tuberculosis* complex in new world monkeys in Peru. Ecohealth.

[31] Wolf T.M., Mugisha L., Shoyama F.M., O’Malley M.J., Flynn J.L., Asiimwe B., Travis D.A., Singer R.S., Sreevatsan S. (2015). Noninvasive test for tuberculosis detection among primates. Emerg. Infect. Dis..

[32] Thomas J., Balseiro A., Gortázar C., Risalde M.A. (2021). Diagnosis of tuberculosis in wildlife: A systematic review. Vet. Res..

[33] Gippoliti S., D’Alessandro A. (2013). Great apes in the giardino zoologico of rome (1910–1998): An overview. Der Zool. Gart..

[34] Prince-Hughes D. (2001). Gorillas among us. A Primate Ethnographer’s Book of Days.

[35] Fiennes R.N.T.W. (1966). The zoological society of London report of the society’s pathologist for the year 1963. J. Zool..

[36] Graham-Jones O. (2001). Zoo Tails.

[37] McCracken H. Gamma Interferon Enzyme Immunoassays and Their Use in the Investigation of Tuberculosis in a Western Lowland Gorilla. Proceedings of the American Association of Zoo Veterinarians.

[38] Michel A.L., Venter L., Espie I.W., Coetzee M.L. (2003). *Mycobacterium tuberculosis* infections in eight species at the National Zoological Gardens of South Africa, 1991–2001. J. Zoo. Wildl. Med..

[39] Scanga C.A., Flynn J.L. (2014). Modeling tuberculosis in nonhuman primates. Cold Spring Harb. Perspect. Med..

[40] Shin N.S., Kwon S.W., Han D.-H., Bai G.-H., Yoon J., Cheon D.S., Son Y.S., Ahn K., Chae C., Lee Y.S. (1995). *Mycobacterium tuberculosis* infection in an orangutan (*Pongo pygmaeus*). J. Vet. Med. Sci..

[41] Son Y.-S., Min K., Cheon D.-S., Chae C. (1996). *Mycobacterium tuberculosis* infection in an orangutan (*Pongo pygmaeus*). Vet. Pathol..

[42] Philippa J., Dench R., Miller R.E., Lamberski N., Calle P.P. (2019). Infectious diseases of orangutans in their home ranges and in zoos. Fowler’s Zoo and Wild Animal Medicine Current Therapy.

[43] Wolf T.M., Sreevatsan S., Travis D., Mugisha L., Singer R.S. (2014). The risk of tuberculosis transmission to free-ranging great apes. Am. J. Primatol..

[44] Calvignac-Spencer S., Leendertz S.A., Gillespie T.R., Leendertz F.H. (2012). Wild great apes as sentinels and sources of infectious disease. Clin. Microbiol. Infect..

[45] Hassell J.M., Zimmerman D., Cranfield M.R., Gilardi K., Mudakikwa A., Ramer J., Nyirakaragire E., Lowenstine L.J. (2017). Morbidity and mortality in infant mountain gorillas (*Gorilla beringei beringei*): A 46-year retrospective review. Am. J. Primatol..

[46] Patrono L.V., Pléh K., Samuni L., Ulrich M., Röthemeier C., Sachse A., Muschter S., Nitsche A., Couacy-Hymann E., Boesch C. (2020). Monkeypox virus emergence in wild chimpanzees reveals distinct clinical outcomes and viral diversity. Nat. Microbiol..

[47] Lonsdorf E.V., Travis D.A., Raphael J., Kamenya S., Lipende I., Mwacha D., Collins D.A., Wilson M., Mjungu D., Murray C. (2021). The gombe ecosystem health project: 16 years of program evolution and lessons learned. Am. J. Primatol..

[48] Hockings K.J., Mubemba B., Avanzi C., Pleh K., Düx A., Bersacola E., Bessa J., Ramon M., Metzger S., Patrono L.V. (2021). Leprosy in wild chimpanzees. Nature.

[49] Williams J.M., Lonsdorf E.V., Wilson M.L., Schumacher-Stankey J., Goodall J., Pusey A.E. (2008). Causes of death in the Kasekela chimpanzees of Gombe National Park, Tanzania. Am. J. Primatol..

[50] Terio K.A., Kinsel M.J., Raphael J., Mlengeya T., Lipende I., Kirchhoff C.A., Gilagiza B., Wilson M.L., Kamenya S., Estes J.D. (2011). Pathologic lesions in chimpanzees (*Pan trogylodytes schweinfurthii*) from Gombe National Park, Tanzania, 2004–2010. J. Zoo. Wildl. Med..

[51] Muhangi D., Gardiner C.H., Ojok L., Cranfield M.R., Gilardi K.V.K., Mudakikwa A.B., Lowenstine L.J. (2021). Pathological lesions of the digestive tract in free-ranging mountain gorillas (*Gorilla beringei beringei*). Am. J. Primatol..

[52] Medkour H., Castaneda S., Amona I., Fenollar F., André C., Belais R., Mungongo P., Muyembé-Tamfum J.J., Levasseur A., Raoult D. (2021). Potential zoonotic pathogens hosted by endangered bonobos. Sci. Rep..

[53] Wolf T.M., Sreevatsan S., Singer R.S., Lipende I., Collins A., Gillespie T.R., Lonsdorf E.V., Travis D.A. (2016). Noninvasive tuberculosis screening in free-living primate Populations in Gombe National Park, Tanzania. Ecohealth.

[54] Hill K., Boesch C., Goodall J., Pusey A., Williams J., Wrangham R. (2001). Mortality rates among wild chimpanzees. J. Hum. Evol..

[55] Lowenstine L.J., McManamon R., Terio K.A. (2016). Comparative pathology of aging great apes: Bonobos, chimpanzees, gorillas, and orangutans. Vet. Pathol..

[56] Formenty P., Boesch C., Wyers M., Steiner C., Donati F., Dind F., Walker F., Le Guenno B. (1999). Ebola virus outbreak among wild chimpanzees living in a rain forest of Côte d’Ivoire. J. Infect. Dis..

[57] Leendertz F.H., Ellerbrok H., Boesch C., Couacy-Hymann E., Mätz-Rensing K., Hakenbeck R., Bergmann C., Abaza P., Junglen S., Moebius Y. (2004). Anthrax kills wild chimpanzees in a tropical rainforest. Nature.

[58] Bermejo M., Rodríguez-Teijeiro J.D., Illera G., Barroso A., Vilà C., Walsh P.D. (2006). Ebola outbreak killed 5000 gorillas. Science.

[59] Leendertz F.H., Lankester F., Guislain P., Néel C., Drori O., Dupain J., Speede S., Reed P., Wolfe N., Loul S. (2006). Anthrax in western and central african great apes. Am. J. Primatol..

[60] Köndgen S., Kühl H., N’Goran P.K., Walsh P.D., Schenk S., Ernst N., Biek R., Formenty P., Mätz-Rensing K., Schweiger B. (2008). Pandemic human viruses cause decline of endangered great apes. Curr. Biol..

[61] Keele B.F., Jones J.H., Terio K.A., Estes J.D., Rudicell R.S., Wilson M.L., Li Y., Learn G.H., Beasley T.M., Schumacher-Stankey J. (2009). Increased mortality and AIDS-like immunopathology in wild chimpanzees infected with SIVcpz. Nature.

[62] Ryan S.J., Walsh P.D. (2011). Consequences of non-intervention for infectious disease in African great apes. PLoS ONE.

[63] Scully E.J., Basnet S., Wrangham R.W., Muller M.N., Otali E., Hyeroba D., Grindle K.A., Pappas T.E., Thompson M.E., Machanda Z. (2018). Lethal respiratory disease associated with human rhinovirus C in wild chimpanzees, Uganda, 2013. Emerg. Infect. Dis..

[64] Negrey J.D., Reddy R.B., Scully E.J., Phillips-Garcia S., Owens L.A., Langergraber K.E., Mitani J.C., Emery Thompson M., Wrangham R.W., Muller M.N. (2019). Simultaneous outbreaks of respiratory disease in wild chimpanzees caused by distinct viruses of human origin. Emerg. Microbes Infect..

[65] Boesch C. (2008). Why do chimpanzees die in the forest? The challenges of understanding and controlling for wild ape health. Am. J. Primatol..

[66] Grützmacher K., Keil V., Leinert V., Leguillon F., Henlin A., Couacy-Hymann E., Köndgen S., Lang A., Deschner T., Wittig R.M. (2018). Human quarantine: Toward reducing infectious pressure on chimpanzees at the Taï Chimpanzee Project, Côte d’Ivoire. Am. J. Primatol..

[67] Michel A.L., Hlokwe T.M., Espie I.W., van Zijll Langhout M., Koeppel K., Lane E. (2013). *Mycobacterium tuberculosis* at the human/wildlife interface in a high TB burden country. Transbound. Emerg. Dis..

[68] Parsons S.D., Gous T.A., Warren R.M., de Villiers C., Seier J.V., van Helden P.D. (2009). Detection of *Mycobacterium tuberculosis* infection in chacma baboons (*Papio ursinus*) using the QuantiFERON-TB gold (in-tube) assay. J. Med. Primatol..

[69] Stephens N., Vogelnest L., Lowbridge C., Christensen A., Marks G.B., Sintchenko V., McAnulty J. (2013). Transmission of *Mycobacterium tuberculosis* from an Asian elephant (*Elephas maximus*) to a chimpanzee (*Pan troglodytes*) and humans in an Australian zoo. Epidemiol. Infect..

[70] Adeogun A., Omobowale O., Owuamanam C., Alaka O., Taiwo V., van Soolingen D., Cadmus S. (2016). *Mycobacterium tuberculosis* and dual *M. tuberculosis*/*M. bovis* infection as the cause of tuberculosis in a gorilla and a lioness, respectively, in Ibadan zoo, Nigeria. Case Rep. Vet. Med..

[71] Kumar S., Laurence H., Owston M.A., Sharp R.M., Williams P., Lanford R.E., Hubbard G.B., Dick E.J. (2017). Natural pathology of the captive chimpanzee (*Pan troglodytes*): A 35-year review. J. Med. Primatol..

[72] Zhou Z., van Hooij A., Vervenne R., Sombroek C.C., Tjon Kon Fat E.M., Ottenhoff T.H.M., Corstjens P.L.A.M., Verreck F., Geluk A. (2021). Quantitative rapid test for detection and monitoring of active pulmonary tuberculosis in nonhuman primates. Biology.

[73] Schliesser T.A., Montali R.J. (1978). Prevalence of tuberculosis and mycobacterial diseases in some European zoos. Mycobacterial Infections of Zoo Animals.

